# An Examination of Bacterial Contamination of Models Used in Anatomy Laboratories

**DOI:** 10.1155/2018/9201312

**Published:** 2018-12-19

**Authors:** Rengin Kosif, Fatma Avcioglu

**Affiliations:** ^1^Anatomy Department, Faculty of Medicine, Bolu Abant Izzet Baysal University, Turkey; ^2^Microbiology Department, Faculty of Medicine, Bolu Abant Izzet Baysal University, Turkey

## Abstract

**Background:**

Bacterial, viral, and parasitic transmission is a common issue involving items that are used in crowded places and are touched. In this study, it was aimed to identify the types of bacteria on models used in anatomy laboratories and the types of bacteria that contaminate students' hands.

**Methods:**

Swab samples were taken from 30 models used in the laboratory and from the dominant hands of 94 students prior to and after contact with the models and were examined in the microbiology laboratory.

**Results:**

Five types of bacteria were isolated from the anatomy models:* coagulase-negative staphylococcus, staphylococcus aureus, bacillus *spp*., enterococcus *spp., and* escherichia coli*.* Coagulase-negative staphylococcus, staphylococcus aureus*, and* bacillus *spp. were isolated from the hands of the students before the contact, and additionally,* enterococcus *spp. were isolated after the contact. The hands were not found to be contaminated with* escherichia coli *originating from the models, whereas* enterococcus *spp. were found to be transmitted to the hands after the contact.

**Conclusion:**

The necessity of washing hands before and after working on the models and the necessity of occasionally disinfecting the models have emerged.

## 1. Introduction

Anatomy models are used in anatomy laboratories as training materials. Models used in training in the Anatomy Department of the Faculty of Medicine at Bolu Abant Izzet Baysal University (BAIBU) were bought in 2004. Since then, the models have been neither washed nor disinfected. Students examine the models by holding them by their hands in the laboratory. Items used in crowded environments and touched by hand are exposed to bacterial, viral, and parasitic transmission [1].

Schultz et al. have researched the bacterial contamination of computer keyboards [2]. Stauber et al. have investigated the bacterial contamination of household toys [3]. Gerba et al. have investigated the bacterial contamination of labels on meat products [4]. Nworie et al. have investigated the bacterial contamination of door handles [1]. Kuhu et al. have investigated the bacterial contamination of stethoscopes [5]. Ngonda has investigated the bacterial contamination of door handles of hospital rooms and restrooms [6].

In this study, it was aimed to investigate the bacterial transmission in anatomy models used in a crowded environment and to show the types and percentage distributions of bacteria. It was also investigated whether or not there were transmissions to the hands of students after they had worked on the models. No article could be found in the literature previously examining the bacterial transmission in anatomy models.

## 2. Materials and Methods

Permission was received for this study from the BAIBU clinical ethics committee. Because the urogenital system subjects were being taught, samples of our study were taken from models of the urogenital system. A total of 94 Grade II students of BAIBU Faculty of Medicine voluntarily participated in the study.

### 2.1. Formation of the Sample Group

A total of 30 models were included in the study from among the urogenital system models used in the Anatomy Practice Laboratory of Faculty of Medicine at Bolu Abant Izzet Baysal University, and 94 volunteer students using these models were included.

Swab samples were taken from the dominant hand of the students prior to contact with the models. Prior to the contact, the students' hands were washed with soap for at least 60 seconds. By this, it was aimed to completely remove the visible dirt on the hands and the temporary flora elements in the skin. Hand swab samples were taken after handwashing from the hands of 30 students, who were considered as the control group, to detect bacteria before working with the models. These samples were cultivated in 3 separate media: blood agar, EMB agar, and chocolate agar.

Next, they were allowed to spend one and a half hours in the laboratory with the selected anatomy models. After one and a half hours of laboratory practice, a hand swab sample was taken once again from the same hand of each student contacting with the models. For this stage, the 94 students were asked to wash their hands before the study, and samples were taken from their hands after spending an hour and a half with the models. Swab samples were also taken from randomly selected 4 cm^2^ surface areas of the 30 models. While swab samples were taken from the models and from the students' hands, swabs soaked with sterile physiological saline solution were used.

### 2.2. Isolation and Identification of Microorganisms

Swab samples were taken from randomly selected 4 cm^2^ surface areas of the models and from the students' hands using swabs soaked with sterile physiological saline solution. These samples were put into 4 ml brain-heart infusion (Brain Heart Broth, Merck) liquid media and sent to our laboratory. Liquid media were incubated at 37°C for 48 hours in aerobic conditions. Then, the samples that were taken by using a 0.001-ml inoculation loop were each cultivated as a single colony in 5% SBA (Sheep Blood Agar), EMB agar (Eosin Methylene Blue), and chocolate agar and incubated again for 48 hours at 37°C. Conventional laboratory methods were used to identify microorganisms reproducing in the media.

After these procedures, some of the colonies reproducing in 5% SBA were first subjected to gram staining to identify gram-positive bacteria. Some of the colonies that were found to be gram-positive were planted in MSA (Mannitol Salt Agar, BD). A 3% H_2_O_2_ catalase test was carried out on the colonies that formed yellow color in MSA, and lambda and tube coagulase tests were also carried out on these colonies. Gram-positive, catalase-positive, and coagulase-positive isolates were considered to be* staphylococcus aureus*. Gram-positive, catalase-positive, and coagulase-negative isolates that reproduced in 5% SBA were considered to be coagulase-negative staphylococcus (CNS). The pyrrolidonyl arylamidase (PYR) test (Becton Dickinson, USA) was applied to the colonies that were negative for the H_2_O_2_ catalase test. Consequently, the PYR-positive isolates were identified as enterococcus. Samples were cultured in EMB agar to identify gram-negative bacteria. Once the microorganisms reproducing in the culture were purified, the species were identified by administering an IMViC (Indole, Methyl red, Voges-Proskauer, Citrate) test and an oxidase test (Becton Dickinson, USA) to the strains with gram- negative reaction based on the gram staining. In the methodology of the study, methods used by O.C. Smibert [7] and Kausar Malik [8] were used.

## 3. Results

**Figure 1:**
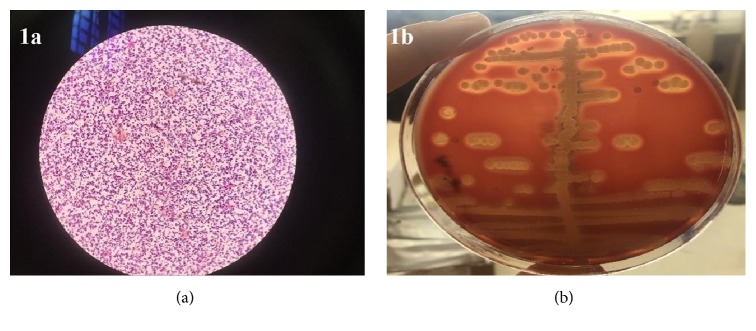
Appearance of Gram's staining of S. aureus detected in the models under the light microscope (×100) (1a) and its beta hemolytic colony appearance in SBA (1b).

A bacterial contamination was observed in all samples cultured from the 30 anatomy models. A total of 19 of the models created skin flora first (coagulase-negative staphylococcus). Potential pathogenic bacteria reproduced in most of the 30 models; specifically, 3 (10%) of the models contained* S. aureus*, 18 (60%) contained* Bacillus *species (spp.), 1 (3%) contained* Enterococcus *spp., and 1 (3%) contained E*scherichia (E.) coli *(Table 1) (Figure 1). A multibacterial colonization was observed in 12 of the models.

It was investigated which bacteria were present on the hands of the students before and after the contact with the models. Differences were found between the bacteria detected on the hands of the students before and after the contact.

Prior to the contact,* coagulase-negative staphylococci*, which are skin flora components,* bacillus *spp., and* staphylococcus aureus *were detected on 23 (77%), 5 (17%), and 2 (7%) of the 30 hands, respectively (Table 2).

**Figure 2:**
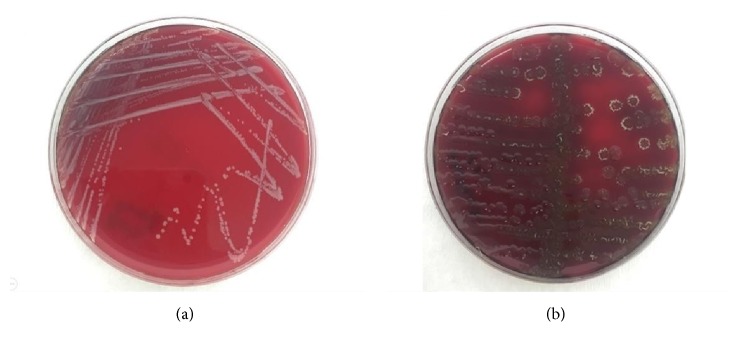
The colony appearance of coagulase-negative staphylococci (2a) and the appearance of bacillus spp. (2b) in SBA after contact.

**Figure 3:**
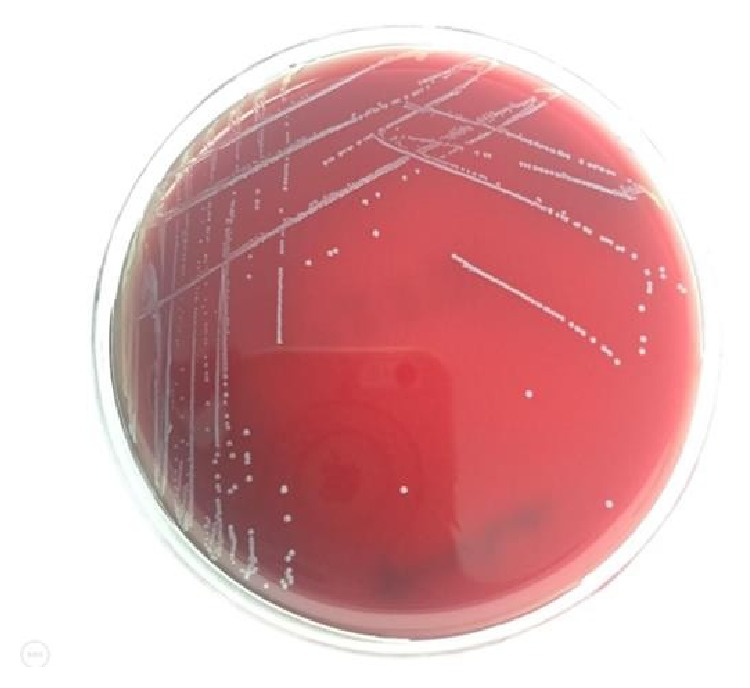
Enterococcus spp. reproducing after contact.

A bacterial colonization was detected in all of the 94 students from whom swab samples were taken after the contact. A multibacterial colonization was present in 47 of them. Mostly coagulase-negative bacterial strains of the skin flora were detected in 77 of the samples taken from the hands after the contact. Potential pathogenic bacteria containing* S. aureus*,* coagulase-negative staphylococci*,* bacillus *spp., and* enterococcus *spp. reproduced in 13 (14%), 77 (82%), 48 (51%), and 3 (3%) of the 94 hand swab samples, respectively (Table 3) (Figures 2 and 3).

While Enterococcus spp. were not observed on the hands before the contact, it was attention grabbing that they colonized on the hands after the contact with the models (Figure 3).

## 4. Discussion

A total of 235 bacteria were isolated from the items used in the hospital, and 98.7% of them consisted of gram-positive bacteria. In a study on bacterial contamination, samples were taken from computers, telephones, curtains, jackets, and neckties [9]. Probes of ultrasound devices and gel covers were examined in terms of bacterial contamination; and staphylococcus aureus, kocuria kristinae, corynebacterium species, and bacillus species were detected [10]. Coagulase-negative staphylococci, staphylococcus aureus, pseudomonas species, and bacillus species reproduced in dentistry clinics [11]. Patient files in surgical intensive care units and other services were examined in terms of bacterial contamination; pathogenic or potential pathogenic bacteria were detected, and most commonly coagulase-negative staphylococcus reproduced [12].

No studies have investigated bacteria on models in anatomy laboratories. The bacteria that reproduced most commonly in our study were coagulase-negative staphylococci, followed by staphylococcus aureus, bacillus spp.,* escherichia coli*, and enterococcus spp. After the laboratory practice, staphylococcus aureus, coagulase-negative staphylococcus, bacillus spp., and enterococcus spp. were detected on the hands of the students. And, enterococcus spp. were found to reproduce as species different from the ones prior to the contact.

Staphylococcus aureus can lead to many local and common diseases. It can cause skin and soft tissue infections, wound infections, deep tissue infections, myositis, pericarditis, endocarditis, pneumonia, osteomyelitis, abscess, and foreign body infections such as shunt, implant, and catheter infections [13]. Coagulase-negative staphylococci cause many secondary infections in humans, are found in medical devices, cause infection in immunosuppressive and other medical treatment areas, and can even cause endocarditis [14].

Bacillus spp. are bacteria that rarely cause acute infection and are frequently isolated in cultures. They can cause local ocular infections associated with trauma, food poisoning, deep soft skin infections, and systemic infections (meningitis, endocarditis, osteomyelitis, and bacteremia). They can cause* Bacillus *(B.)* subtilis*,* B. sphaericus*,* B. alvei*,* B. laterosporus*,* B. licheniformis*,* B. megaterium, *and* B. pumilus*, the most common of them being* B. cereus *[15].

Enterococcus spp. cause infections of digestive system, urinary system, abdominal organs, and surgical wounds. They can spread to the endocardium from there. They are resistant to many antibiotics. Enterococci can cause infections in and out of hospitals. These bacteria have the ability to stick to cardiac valves and kidney epithelial cells [16]. Enterococci are normally very resistant to environmental conditions. It can keep alive for a long time in outdoor environments [17, 18]. The presence of a small amount of enterococci (3%) in the models and the interaction of the enterococci in the samples taken from the hands after the students' contact with the models show that the contamination is caused by the models. Since we have been using models for a long time in our medical faculty and have not been cleaned up until now, we can be contaminated by bacteria that can maintain its vitality for a long time while working with models. This shows us that students and laboratory staff should pay more attention to hand hygiene than normal.

*Escherichia coli *is a common cause of digestive system infections, urinary system infections, bacteremia, cholecystitis, and acute bacterial meningitis through the consumption of contaminated water and nutrients. Although it is harmless in healthy human intestines, certain pathogenic strains of it can also cause diarrhea, peritonitis, mastitis, septicemia, hemolytic uremic syndrome, respiratory infections, and pneumonia [19].

*E. coli* is a bacterium of the normal flora, mostly found in the gastrointestinal tract. In particular, detecting the presence of bacteria in the water indicates fecal contamination [20]. In our study, 3%* E. coli* have been detected in the models and this condition proves the presence of fecal contamination. This has revealed the requirement of regular cleaning of the models.

In this study, the contamination on the students' hands after the contact with the models was worse compared to the condition prior to the contact, and s. aureus rose from 7% to 14%, CNS rose from 77% to 88%, and bacillus spp. rose from 17% to 51%. The greatest rise was in bacillus spp.* E. coli *was not transmitted to the hands. Even though the hands were washed prior to contact with the models, not all of the bacteria found on the hands were destroyed. It was observed that the rates of bacteria on the hands were increased as a result of the exposure to the models for one and half hours. It was understood based on these results that hands must be washed before the contact with models as well as after the contact.

The pathogenic bacteria that grow on models used in laboratories are staphylococcus aureus,* E. coli*, and enterococcus. CNS and bacillus spp. can be pathogenic since they are resistant to antibiotics. It was found that the students' hands were infected with these bacteria during the study.

Five types of bacteria were isolated from the anatomy models:* coagulase-negative staphylococcus, staphylococcus aureus, bacillus *spp*., enterococcus *spp., and* escherichia coli*. Students and all faculty working in anatomy laboratories should be ensured to stay away from bacteria by having them wash their hands with soap for at least 120 seconds before and after laboratory hours. As models also contain potentially pathogenic bacteria, the necessity of disinfecting them at specific intervals has arisen.

## Figures and Tables

**Table 1:** Bacterial species and rates detected in all models.

	Staphylococcus aureus (%)	Coagulase Negative Staphylococcus (%)	Bacillus spp. (%)	Escherichia coli (%)	Enterococcus spp. (%)
Models	3/30 (10%)	19/30 (63%)	18/30 (60%)	1/30 (3%)	1/30 (3%)

**Table 2:** Bacterial species and rates detected on the hands of 30 students before contact.

	Staphylococcus aureus (%)	Coagulase Negative Staphylococcus (%)	Bacillus spp. (%)
Hands before the contact	2/30 (7%)	23/30 (77%)	5/30 (17%)

**Table 3:** Bacterial species and rates detected on the hands after contact.

	Staphylococcus aureus (%)	Coagulase Negative Staphylococcus (%)	Bacillus spp. (%)	Enterococcus spp. (%)
Hands after the contact	13/94 (14%)	77/94 (82%)	48/94 (51%)	3/94 (3%)

## Data Availability

The data used to support the findings of this study have been deposited at Dr. Fatma Avcioglu, fatmaavcioglu@yahoo.com.tr. The samples data used to support the findings of this study are included within the article. Requests for data [6/12 months] after publication of this article will be considered by the corresponding author (Dr. Rengin Kosif, rengink@yahoo.com). The samples data used to support the findings of this study are restricted by the BAIBU Clinical Ethics Board. The data used to support the findings of this study are available from the corresponding author upon request.
